# The close relationship between inflammation and insulin resistance: a comparative perspective from a new angle

**DOI:** 10.1007/s00592-025-02596-y

**Published:** 2025-10-15

**Authors:** Xiaoyan Wang, Run Yang, Jingxiang Li, Yongqi Liang, Chenxi Jin, Yining Xu, Xianbo Wu, Mengchen Zou

**Affiliations:** 1 Department of Endocrinology and Metabolism, Nanfang Hospital, Southern Medical University, 1838 Guangzhou Road North, Guangzhou, 510515 China; 2 Department of Occupational Health and Medicine, School of Public Health, Southern Medical University, Guangzhou, China

**Keywords:** Excess weight, Inflammation, Insulin resistance, Physically active

## Abstract

**Background:**

Excess weight is a progressive metabolic epidemic, and inflammation plays an important role in the progression of disease. Insulin resistance (IR) is an important feature of obesity, but it does not reflect systemic inflammation. Currently, there is a lack of *effective* clinical tools for early risk stratification and intervention in physically active people.

**Methods:**

This was a prospective cohort of 72,262 overweight but physically active persons in the UK Biobank. The TyG was combined with hsCRP, waist circumference (WC), or body mass index (BMI) as indices of IR. Adjusted Cox regression, interaction tests, restricted cubic splines (RCS) analysis, Kaplan-Meier analysis, and Harrell’s C-index were used to examine the relations and time-dependent predictive power.

**Results:**

During 12.7 years of follow-up, 1,477 participants developed metabolic dysfunction-associated fatty liver disease (MAFLD). RCS analysis suggested TyG-hsCRP had a nonlinear positive correlations with all-cause mortality. Compared to the lowest quartile group, the corrected hazard ratio (HR) (95% confidence interval [CI]) of new-onset MAFLD in maximum quartile groups for TyG-hsCRP was 1.94(1.62–2.32), for TyG-WC was 1.78(1.44–2.18), for TyG-BMI was 1.36(1.12–1.65), and for TyG was 1.41(1.15–1.72). The relation between C-index of TyG-hsCRP and MAFLD was higher than that of other TyG indices. Similar results were observed in all-cause mortality.

**Conclusion:**

TyG-hsCRP is superior to other indices for identifying risk of MAFLD and all-cause mortality in overweight but physically active people. Our findings suggest the importance of inflammatory metabolism and provide evidence for effectively early anti-inflammatory treatments.

**Supplementary Information:**

The online version contains supplementary material available at 10.1007/s00592-025-02596-y.

## Introduction

Excess weight is a complex metabolic condition associated with metabolic dysfunction-associated fatty liver disease (MAFLD), cardiovascular events, cancer, and all-cause mortality, affecting about 2 billion people worldwide and resulting in a huge economic burden [1]. Inflammation is a key driver of disease development in individuals with excess weight [2, 3], but high-intensity exercise may reverse the metabolic damage caused by chronic inflammation [4]. Many professional expert associations consider lifestyle changes, such as increased exercise and diet restriction, to be the cornerstone of obesity management [5, 6]. Many studies have analyzed the relations between body mass index (BMI) and metabolic phenotype with disease development [7], as well as the changes in metabolic phenotypes over time [8]. Although the criteria for metabolic syndrome have been established, they are still evolving, resulting in a lack of specific guidelines, which makes clinical intervention somewhat difficult to initiate [9]. Few studies have focused on the use of specific metabolic markers, such as insulin resistance (IR) and inflammation, to stratify high-risk populations and initiate early interventions in individuals with excess weight.

The triglyceride-glucose (TyG) index is a generally recognized indicator of IR. When combined with BMI and waist circumference (WC), TyG and related indices have been shown to be predictive of the development of MAFLD [10, 11]. On the other hand, studies have reached inconsistent conclusions regarding the optimal index for predicting the risk of MAFLD or mortality [12, 13]. Given that inflammation plays an important role in obesity and metabolism, and is affected by exercise [14], we believe existing assessments do not take into account inflammation. The TyG index and hsCRP have been combined into a composite index for predicting the prognosis of patients with malignancies [15]. However, the application of this composite indicator in other populations remains unclear and the selection of inflammatory markers is still relatively limited in existing literature due to the absence of standardized calculation formulas.

Based on the previous exploration of the TyG index, we performed a combined analysis of TyG and hsCRP, comparing the predictive ability with earlier indicators. Our indicators encompass more than just IR and inflammation, allowing for direct comparison with TyG-WC and TyG-BMI. Given that individuals who exercise regularly are more likely to maintain a healthy weight and improve their metabolism, receiving early clinical guidance could potentially enhance their health outcomes.

## Methods

### Research design and methods

The UK Biobank (UKB) is a large prospective database that collected detailed information of 502,507 participants recruited between 2006 and 2010 [13]. Each participant filled out questionnaires on demographic characteristics, lifestyle habits, health status, and finished related physical examinations, blood biochemical testing. This study excluded participants who did not engage in regular physical activity, had a BMI outside the range of 25–29.9 kg/m^2^ [2], and who had been diagnosed with MAFLD prior to enrollment (regular physical activity: at least 150 min/week of moderate activity [50%-70% of the maximum heart rate after exercise], or 75 min/week of vigorous activity [70%-85% of the maximum heart rate after exercise], or an equivalent combination. Maximum heart rate: 220 minus your age [in years] [11]). In addition, participants with missing key data were excluded. Finally, 72,262 participants were were included (Fig. S1).

### Outcomes

The primary outcome of this study was MAFLD. By using the International Classification of Diseases, 10th revision (ICD-10) and the Expert Panel Consensus Statement, MAFLD was defined as ICD-10 code K76.0 (fatty [change of] liver, not elsewhere classified) and K75.8 (other specified inflammatory liver diseases).

The secondary outcome was all-cause mortality. Death outcomes were identified through linkage to national death registries, and detailed death registration information can be found at link https://biobank.ndph.ox.ac.uk/~bbdatan/DeathSummaryReport.html.

### IR indices and covariates

Fasting plasma glucose (FPG) and triglyceride levels were analyzed using a Hitachi 7180 chemistry analyzer, and hs-CRP was detected by an immunoturbidimetric high-sensitivity assays on a Beckman Coulter AU5800. Height, weight, and WC were physically measured 3 times, and the average values were adopted. BMI was calculated as weight (in kilograms) *divided by* height (in meters) squared. TyG index was calculated as ln [triglycerides (mg/dl)×glucose (mg/dl)/2]. The TyG-hsCRP index was calculated as follows: 0.412×ln (CRP) + TyG. The TyG-WC and TyG-BMI indices were calculated by multiplying TyG by WC or BMI [12].

Information on socio-demographic status and health-related factors was collected using a structured questionnaire. Socio-demographic variables included age, sex (male/female), and race (white/non-white). Health-related lifestyle habits included smoking (never/former/current), drinking (never/former/current), insomnia (rarely/sometimes/often) and cumulative dietary risk score ( a continuous variable, with a score ranging from 0 to 9) (Table S1) [11]. Relevant blood biochemical indicators included glycosylated hemoglobin (HbA1c), high-density lipoprotein cholesterol (HDL-C), low-density lipoprotein cholesterol (LDL-C), total cholesterol, creatinine, urea, alanine aminotransferase (ALT), aspartate aminotransferase (AST), and total bilirubin. The quality check procedure for blood samples is available at https://biobank.ndph.ox.ac.uk/showcase/showcase/docs/biomarker_issues.pdf.

### Statistical analysis

The exposure variables TyG-hsCRP, TyG-WC, TyG-BMI, and TyG were divided into quartiles from lowest (Q1) to highest (Q4). The risk calculation was based on Q1, including hazard ratio (HR), 95% confidence interval (CI) and corresponding p-value. Mean interpolation was used in missing continuous variables, and missing categorical variables were excluded. Continuous variables were expressed as mean and standard deviation (SD), and categorical variables were expressed by frequency and proportion. The trend test was performed using the interval median value. The t test (continuous variable) or chi-square test (categorical variable) were used to assess the differences between subjects grouped by quartiles.

Characteristics of participants were compared based *on* MAFLD. Variables between the 2 groups with a p value < 0.05 were included in subsequent multivariate adjustment models. We made a proportional hazards assumption for the Cox model, and no violations were detected (Fig. S2). In the analysis of TyG correlation index and outcomes, the models were as follows. Model 1 was unadjusted; Model 2 was partially adjusted; Model 3 was fully adjusted. Dose-response relations between TyG-hsCRP and outcomes were evaluated by restricted cubic splines (RCS) regression analysis. Cox proportional hazards models were employed to assess HRs and 95% CIs of TyG correlation index with outcomes. In order to weigh the advantages and disadvantages of each indicator, we calculated the time-dependent Harrell’s C-indices of TyG-hsCRP, TyG-WC, TyG-BMI, and TyG, and we selected the index with the highest predictive power for outcomes and conducted a subsequent primary analysis. In addition, we performed subgroup analyses based on age or sex to further compare C-indices [12]. The interaction of age, sex, ethnicity, smoking, drinking, and sleep in TyG-hsCRP with outcomes was analyzed using additive product terms in the Cox models. The Kaplan-Meier method was used to develop survival curves, and differences in survival were evaluated with a stratified log-rank test and a multivariable Cox proportional-hazards model. To minimize errors in population selection, statistical methods, and variable inclusion, we conducted a comprehensive sensitivity analysis. Statistical analyses was performed using R studio software, version 4.3.2 and p values < 0.05 were considered to indicate statistical significance.

## Results

### Baseline characteristics of participants

The mean age of the 72,262 participants included was 56 ± 8.2 years, with 58% being male. Participants were divided into non-MAFLD and MAFLD, and this was used as a baseline analysis to identify differential variables (*p* < 0.05). Table 1 showed baseline characteristics, and results revealed that participants who developed MAFLD were more likely to be older and have higher BMI, WC, fasting blood glucose, HbA1c, hsCRP, triglycerides, ALT, AST, and total bilirubin (Table 1).

**Table 1:** Baseline characteristics of included participants in the UK biobank

Characteristics	Total	Non-MAFLD	MAFLD	*p*.overall
	*N* = 72,262	*N* = 70,785	*N* = 1477	
Age, years	56.3 (8.23)	56.3 (8.23)	57.6 (7.92)	<0.001
Sex, n(%)				0.201
Female	30,454 (42.1%)	29,807 (42.1%)	647 (43.8%)	
Male	41,808 (57.9%)	40,978 (57.9%)	830 (56.2%)	
Ethnic_group, n(%)				0.013
White	64,517 (89.3%)	63,228 (89.3%)	1289 (87.3%)	
Non-white	7745 (10.7%)	7557 (10.7%)	188 (12.7%)	
Drinking, n(%)				<0.001
Never	2140 (2.96%)	2097 (2.96%)	43 (2.91%)	
Former	1917 (2.65%)	1851 (2.61%)	66 (4.47%)	
Current	68,205 (94.4%)	66,837 (94.4%)	1368 (92.6%)	
Smoking, n(%)				<0.001
Never	66,043 (91.4%)	64,741 (91.5%)	1302 (88.2%)	
Former	3973 (5.50%)	3851 (5.44%)	122 (8.26%)	
Current	2246 (3.11%)	2193 (3.10%)	53 (3.59%)	
Insomnia, n(%)				<0.001
Occasional	20,039 (27.7%)	19,696 (27.8%)	343 (23.2%)	
Sometimes	34,801 (48.2%)	34,089 (48.2%)	712 (48.2%)	
Often	17,422 (24.1%)	17,000 (24.0%)	422 (28.6%)	
Cumulative dietary risk factor score	4.46 (1.45)	4.46 (1.45)	4.53 (1.46)	0.056
BMI, kg/m^2^	27.2 (1.37)	27.2 (1.37)	27.5 (1.40)	<0.001
Waist, cm	90.5 (8.10)	90.5 (8.09)	92.2 (8.21)	<0.001
glucose, mmol/L	5.05 (0.98)	5.05 (0.97)	5.20 (1.30)	<0.001
HbA1c, mmol/mol	35.3 (5.31)	35.3 (5.28)	36.3 (6.52)	<0.001
HbA1c,%	5.31(0.99)	5.28(0.95)	5.52(1.19)	< 0.001
CRP, mg/L	2.16 (3.61)	2.15 (3.59)	2.78 (4.34)	<0.001
HDL, mmol/L	1.43 (0.35)	1.43 (0.35)	1.39 (0.37)	<0.001
LDL, mmol/L	3.65 (0.85)	3.65 (0.85)	3.57 (0.87)	0.001
triglycerides, mmol/L	1.77 (0.98)	1.76 (0.97)	2.00 (1.15)	<0.001
cholesterol, mmol/L	5.78 (1.11)	5.79 (1.11)	5.70 (1.18)	0.003
ALT, U/L	23.7 (12.8)	23.5 (12.5)	30.6 (22.1)	<0.001
AST, U/L	26.5 (9.87)	26.4 (9.47)	31.6 (20.9)	<0.001
TBil, umol/L	9.50 (4.50)	9.49 (4.48)	9.85 (5.38)	0.010
urea, mmol/L	5.50 (1.28)	5.50 (1.28)	5.51 (1.35)	0.794
cr, umol/L	74.8 (15.1)	74.8 (15.1)	73.4 (15.2)	0.001

Glucose: fasting blood glucose; HbA1c: glycosylated hemoglobin; CRP: highly sensitive C-reactive protein; HDL: high-density lipoprotein cholesterol; LDL: low-density lipoprotein cholesterol; triglycerides: triglyceride; cholesterol: total cholesterol; ALT: alanine aminotransferase; AST: aspartate aminotransferase; TBiL: total bilirubin; urea: urea; cr: creatinine;. Continuous variables are presented as the mean and SD, category variables are described as the frequency and percentage

### RCS analysis

**Fig. 1:**
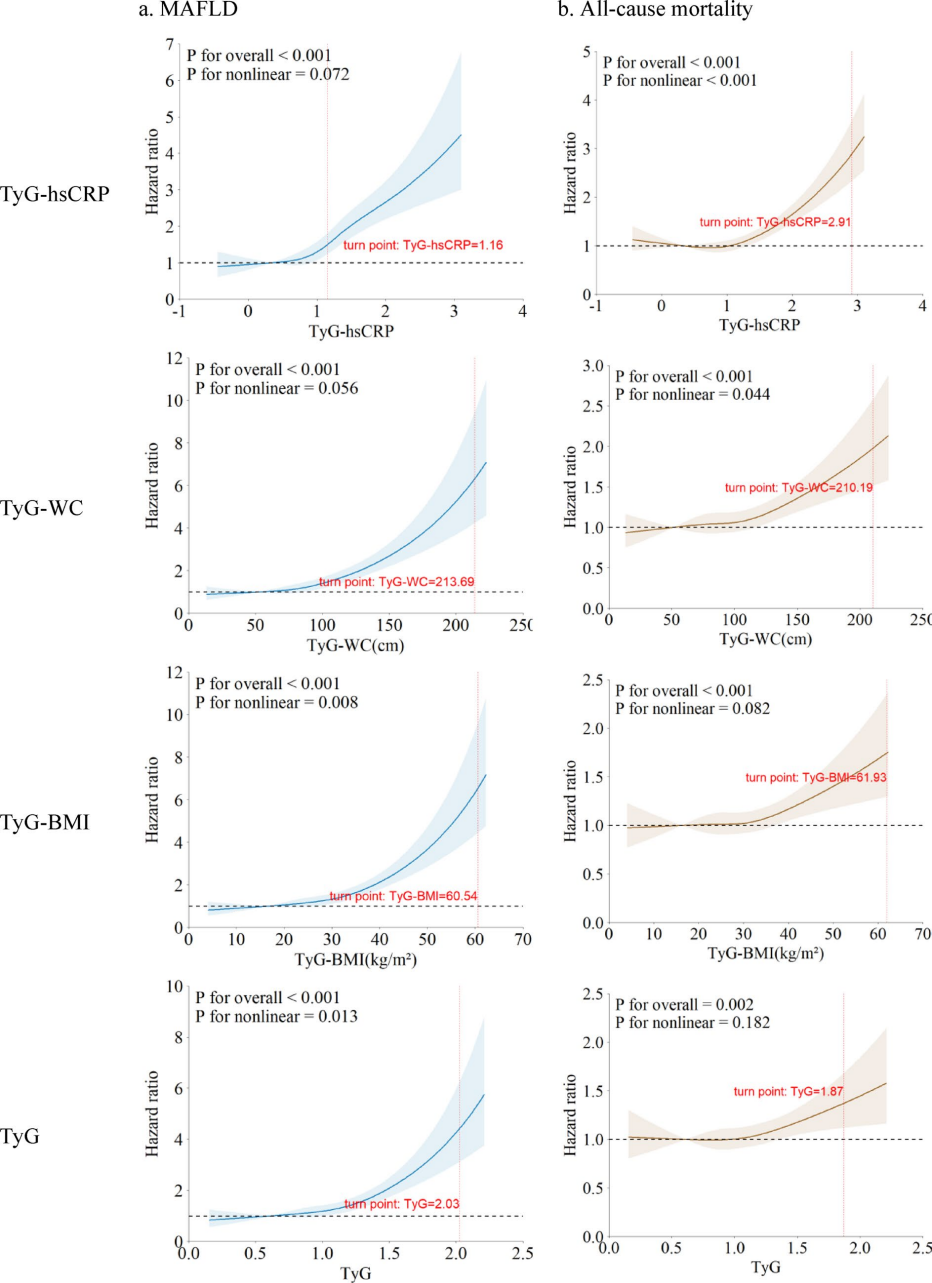
Non‑linear relationships of TyG-hsCRP, TyG-WC, TyG-BMI, and TyG for MAFLD (**a**) and all-cause mortality (**b**). Hazard ratios were adjusted for age and sex. The graph highlights the reference points with significant variations

The RCS analysis showed a nonlinear positive association between TyG-hsCRP and the future risk of all-cause mortality (p for nonlinear < 0.05). The turning points for TyG-hsCRP in MAFLD and all-cause mortality were 1.16 and 2.91, respectively. Similarly, results revealed a nonlinear relationship between TyG-WC and all-cause mortality, TyG-BMI and MAFLD, TyG and MAFLD (Fig. 1).

### Relationship between TyG-hsCRP, TyG-WC, TyG-BMI, and TyG with MAFLD, and all-cause mortality

During the median follow-up period 12.7 years, 1,477 participants (2.0%) had new-onset MAFLD and 4,100 (5.7%) died (all cause). Regarding TyG-hsCRP and MAFLD, in Model 3, the HRs for Q2-4 were 1.31 (1.10–1.56), 1.51 (1.27–1.80), and 1.94 (1.62–2.32), respectively (p for trend < 0.0001) (Table 2). In model 1, the risk of developing MAFLD increased 40% for each SD increase of TyG-hsCRP (HR = 1.40, 95% CI: 1.34–1.47).

**Table 2:** The association between TyG-hsCRP, TyG-WC, TyG-BMI, TyG with MAFLD or all-cause mortality in UK biobank

Characteristic	Crude model (Model 1)	Partially adjusted model (Model 2)	Fully adjusted model (Model 3)
	HR (95% CI)*p* value	HR (95% CI)*p* value	HR (95% CI)*p* value
MAFLD			
TyG-hsCRP			
Quartile 1	1.00(Ref)	1.00(Ref)	1.00(Ref)
Quartile 2	1.44(1.22–1.71)***	1.40(1.18–1.66)***	1.31(1.10–1.56)**
Quartile 3	1.78(1.51–2.10)***	1.71(1.45–2.02)***	1.51(1.27–1.80)***
Quartile 4	2.58(2.21–3.01)***	2.44(2.09–2.86)***	1.94(1.62–2.32)***
*p* for trend	< 0.0001	< 0.0001	< 0.0001
TyG-WC			
Quartile 1	1.00(Ref)	1.00(Ref)	1.00(Ref)
Quartile 2	1.23(1.04–1.44)*	1.22(1.03–1.43)*	1.19(1.01–1.42)*
Quartile 3	1.38(1.18–1.62)***	1.40(1.19–1.65)***	1.35(1.13–1.62)**
Quartile 4	2.02(1.75–2.34)***	2.13(1.82–2.49)***	1.78(1.44–2.18)***
*p* for trend	< 0.0001	< 0.0001	< 0.0001
TyG-BMI			
Quartile 1	1.00(Ref)	1.00(Ref)	1.00(Ref)
Quartile 2	1.18(1.04–1.39)*	1.14(0.97–1.35)	1.15(0.97–1.37)
Quartile 3	1.37(1.18–1.61)***	1.33(1.14–1.56)***	1.27(1.06–1.52)**
Quartile 4	1.94(1.68–1.25)***	1.90(1.64–2.21)***	1.62(1.32–1.99)***
*p* for trend	< 0.0001	< 0.0001	< 0.0001
TyG			
Quartile 1	1.00(Ref)	1.00(Ref)	1.00(Ref)
Quartile 2	1.28(1.09–1.51)**	1.24(1.06–1.46)**	1.20(1.02–1.41)*
Quartile 3	1.45(1.24–1.70)***	1.40(1.24–1.64)***	1.31(1.10–1.56)**
Quartile 4	1.80(1.55–2.10)***	1.76(1.51–2.05)***	1.41(1.15–1.72)***
*p* for trend	< 0.0001	< 0.0001	< 0.0001
All-cause mortality			
TyG-hsCRP			
Quartile 1	1.00(Ref)	1.00(Ref)	1.00(Ref)
Quartile 2	1.28(1.16–1.41)***	1.07(0.97–1.18)	1.06(0.96–1.17)
Quartile 3	1.43(1.30–1.57)***	1.12(1.02–1.24)*	1.11(1.01–1.22)*
Quartile 4	2.05(1.87–2.24)***	1.54(1.41–1.68)***	1.46(1.32–1.62)***
*p* for trend	< 0.0001	< 0.0001	< 0.0001
TyG-WC			
Quartile 1	1.00(Ref)	1.00(Ref)	1.00(Ref)
Quartile 2	1.29(1.17–1.42)***	0.99(0.90–1.09)	0.99(0.90–1.09)
Quartile 3	1.53(1.40–1.68)***	1.08(0.98–1.18)	1.08(0.97–1.20)
Quartile 4	1.74(1.59–1.90)***	1.16(1.05–1.27)**	1.14(1.01–1.29)*
*p* for trend	< 0.0001	< 0.0001	0.01
TyG-BMI			
Quartile 1	1.00(Ref)	1.00(Ref)	1.00(Ref)
Quartile 2	1.29(1.17–1.42)***	0.99(0.90–1.09)	0.99(0.90–1.10)
Quartile 3	1.53(1.40–1.68)***	1.08(0.98–1.18)	1.08(0.97–1.20)
Quartile 4	1.74(1.59–1.90)***	1.16(1.05–1.27)**	1.14(1.01–1.30)*
*p* for trend	< 0.0001	< 0.001	0.91
TyG			
Quartile 1	1.00(Ref)	1.00(Ref)	1.00(Ref)
Quartile 2	1.25(1.14–1.37)***	1.03(0.94–1.13)	1.03(0.93–1.13)
Quartile 3	1.37(1.25–1.50)***	1.06(0.97–1.16)	1.06(0.96–1.17)
Quartile 4	1.51(1.38–1.65)***	1.15(1.05–1.26)**	1.11(0.99–1.25)
*p* for trend	< 0.0001	< 0.0001	0.07

Model 1, no covariates were adjusted

Model 2, age, sex were adjusted

Model 3, general variable: age, sex, race, smoking, drinking, insomnia, HbA1c, HDL, LDL, cholesterol, cr, ALT, AST, totalbilirubin; regarding TyG-hsCRP: general variable, WC, BMI were adjusted; regarding TyG-WC: general variable, BMI, crp were adjusted; regarding TyG-BMI: general variable, WC, crp were adjusted; regarding TyG: general variable, WC, BMI, crp were adjusted

TyG-hsCRP : Q1[-0.70-0.79], Q2[0.79–1.12], Q3[1.12–1.45], Q4[1.45–3.58]

TyG-WC (cm): Q1[-13.3-73.4], Q2[73.4–91.4], Q3[91.4–111], Q4[111–274]

TyG-BMI *(kg/m*^*2*^*)*: Q1[-4.23-22.5], Q2[22.5–27.5], Q3[27.5–32.7], Q4[32.7–74.4]

TyG : Q1[-0.16-0.84], Q2[0.84–1.01], Q3[1.01–1.20], Q4[1.20–2.89]

HR: hazard ratio; CI: confidence interval. * *p* < 0.05, ** *p* < 0.001, *** *p* < 0.0001; *p* < 0.05 was considered statistically significant

Regarding TyG-hsCRP and all-cause mortality, in Model 3, the HRs for Q2-4 were 1.06 (0.96–1.17), 1.11 (1.01–1.22), and 1.46 (1.32–1.62), respectively (p for trend < 0.0001) (Table 2). In model 1, all-cause mortality increased by 32% for each SD increase of TyG-hsCRP (HR = 1.32, 95% CI: 1.28–1.36).

Similar analyses were conducted on the relations between TyG-WC, TyG-BMI, and TyG (Table 2). The results showed in Model 3 TyG-BMI and TyG had no statistically significant association with all-cause mortality (p for trend 0.91 and 0.07, respectively).

### Predictive ability comparison

**Fig. 2:**
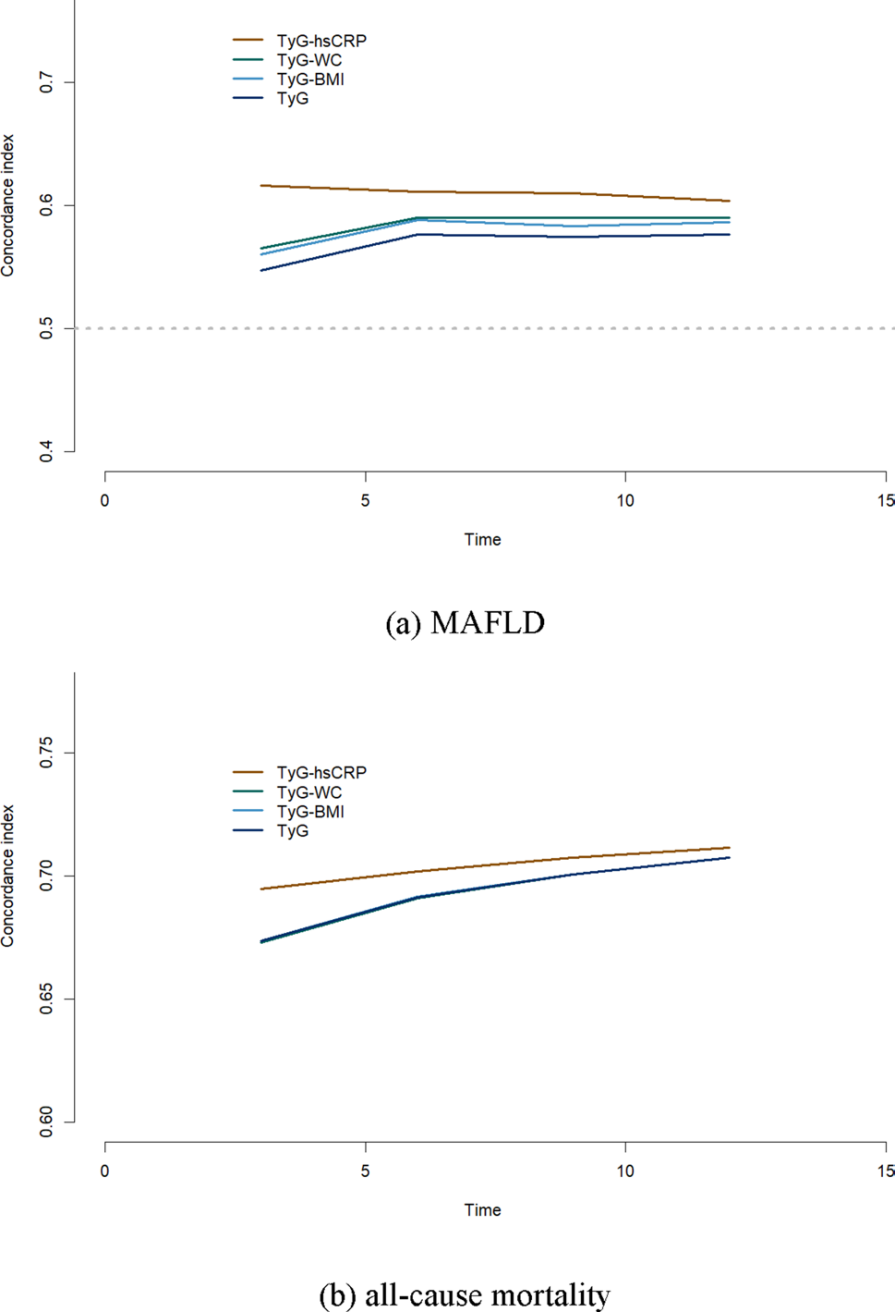
Time-dependent predictive ability of TyG-hsCRP, TyG-WC, TyG-BMI, and TyG for MAFLD (**a**), all-cause mortality (**b**). Models were adjusted for age and sex. Figure legends: Brown lines = group TyG-hsCRP; Green lines = group TyG-WC; Light blue lines = group TyG-BMI; Dark blue line = group TyG

We calculated the time-dependent Harrell’s C-indices, and the results indicated for MAFLD, TyG-hsCRP exhibited the strongest predictive ability, followed by TyG-WC, TyG-BMI, and TyG. In terms of new-onset mortality, TyG-hsCRP again presented the strongest predictive power, while the predictive abilities of TyG-WC, TyG-BMI, and TyG showed no significant differences (Fig. 2). The predictive power in gender subgroups and age subgroups was also compared. The results indicated, with the exception of MAFLD events in individuals over 60 years, where TyG-WC showed superior predictive ability, TyG-hsCRP consistently outperformed in other subgroups (**Fig. S3-4**).

**Fig. 3:**
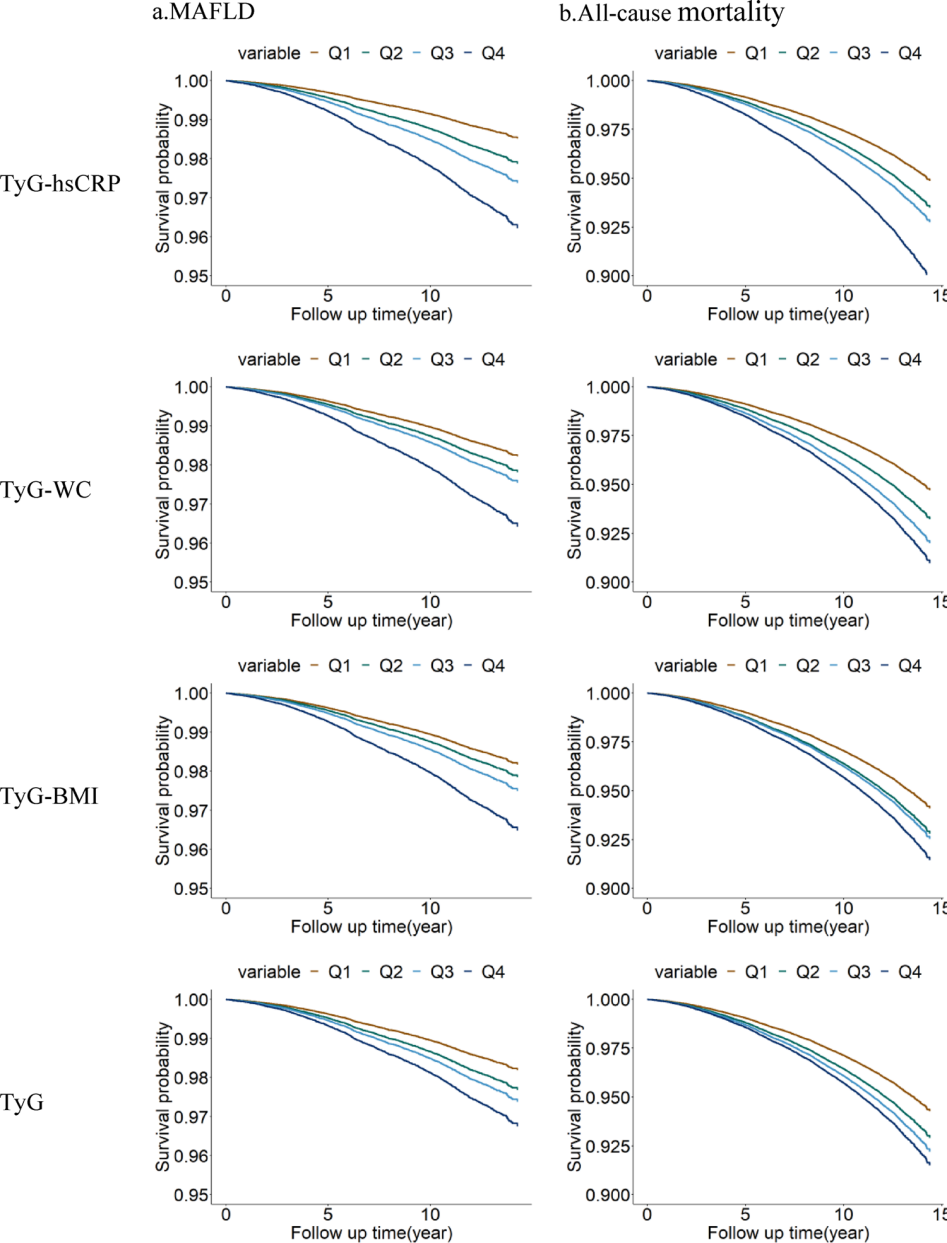
Kaplan-Meier curves of TyG-hsCRP, TyG-WC, TyG-BMI, and TyG for MAFLD (**a**) and all-cause mortality (**b**). Models were adjusted for age and sex. Brown lines = group 1; Green lines = group 2; Light blue lines = group 3; Dark blue line = group 4

**Fig. 4:**
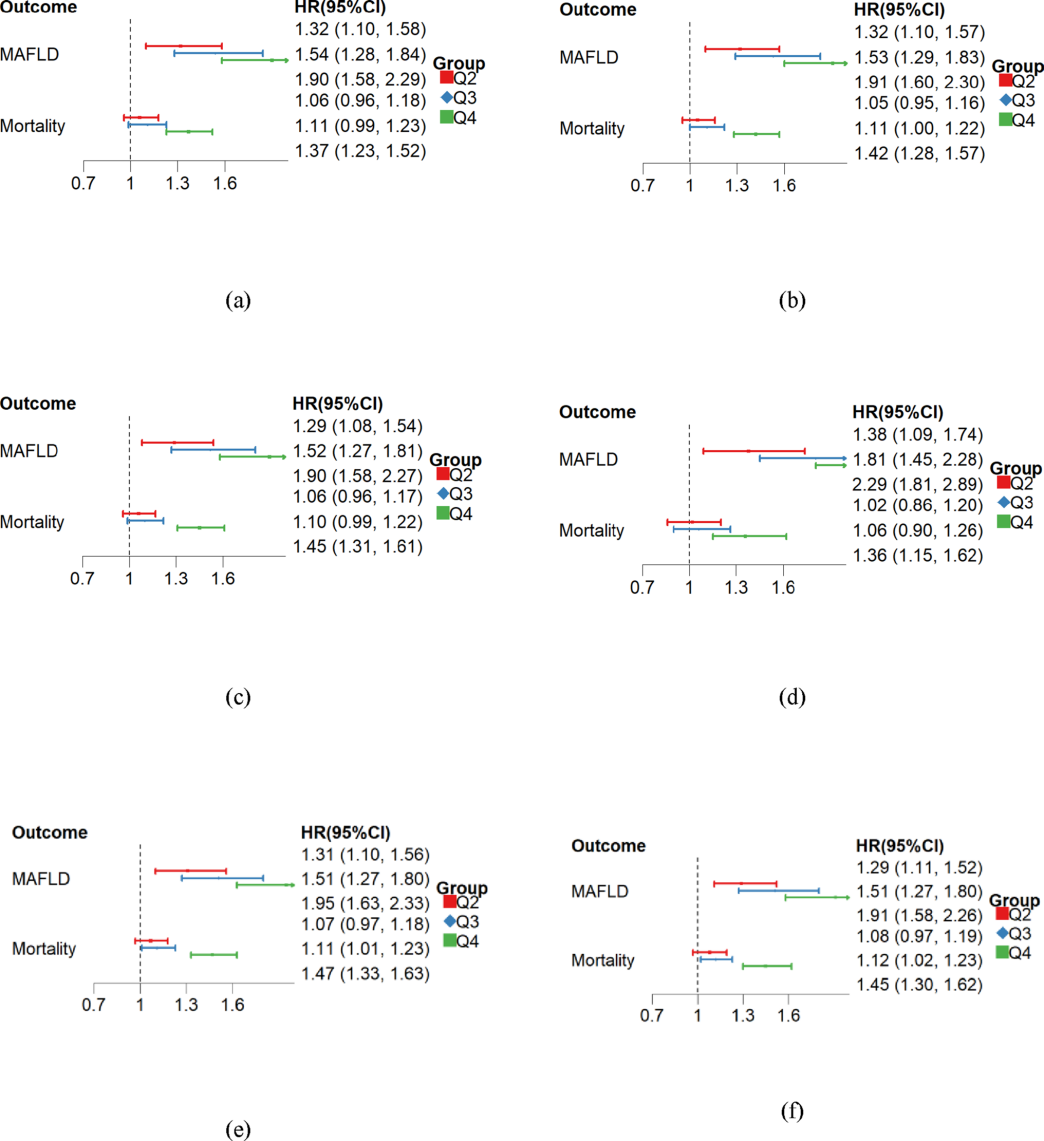
Sensitivity analysis of TyG-hsCRP for predicting MAFLD and all-cause mortality. All differential variables were adjusted for in the model, and the first quartile (Q1) served as the reference interval. **a** Without 603 participants who had a study outcomes within 2 years. **b** Without 1446 participants with extremely low or high TyG-hsCRP levels. **c** Without 695 diabetic participants. d) Without 16,118 participants with malignancies. **e** Multiple imputation replacement of mean imputation. **f** Adjustment for covariates (including white blood cells, hemoglobin, and platelets)

### Kaplan-Meier analysis

The Kaplan-meier curves showed participants with higher TyG-hsCRP levels were more likely to develop MAFLD and all-cause mortality than those with lower TyG-hsCRP levels (log rank test *p* < 0.05). Analyses of TyG-WC, TyG-BMI, and TyG produced similar results (Fig. 3).

### Interaction and subgroup analysis, and sensitivity analysis

The results showed age and drinking had interactions with MAFLD. Age and race had interactions with all-cause mortality. Subgroup analyses indicated people aged 20–60 years were more likely to develop MAFLD or all-cause mortality (**Fig. S5**).

To ensure the robustness of the results, we first excluded 603 participants who experienced an outcome event within 2 years of follow-up. Secondly, we deleted 1,446 cases with the maximum (> 99%) or minimum (< 1%) values of TyG-hsCRP. Thirdly, we excluded 695 people who had diabetes at enrollment to eliminate the potential effect of IR. Fourthly, 16,118 participants with tumors were excluded. Furthermore, we employed multiple imputation instead of mean imputation. Finally, we made adjustments to the selection of covariates (Fig. 4). The results showed the model remained robust.

## Discussion

In this prospective cohort study, we explored the associations of TyG correlation indexes with new-onset MAFLD, all-cause death and compared their time-dependent predictive power in the overall population, and in sex and age subgroups. Overall, the TyG-hsCRP index, rather than TyG-BMI, TyG-WC or TyG, had the highest predictive power, which suggested that metabolic indicators, rather than physical measurement indices, may warrant greater attention. The findings highlight the potential impact of inflammatory factors in the development of the disease, providing evidence for anti-inflammatory therapy. In addition, the effect value was more significant in the young group between 20 and 60 years old. This suggests that we should pay more attention to the young group and implement high-intensity exercise training and clinical intervention, in order to achieve inflammation reversal and improve outcomes.

Obesity has greatly increased the medical burden due to associated long-term systemic metabolic disorders, which contributes to a high incidence of various diseases [16]. Excess weight can lead to low-grade chronic inflammation, IR, liver metabolic disorders, β cell damage, which increases the occurrence of MAFLD, cardiovascular events, and all-cause death [17]. Fortunately, exercise is a lifestyle habit that can effectively reduce the level of inflammation in the body [18], thus offering an opportunity to reverse inflammation and improve health outcomes. Studies have shown that inflammation is an important part in people with excess weight, and macrophages mediate the increase of systemic circulating inflammatory factors, such as CRP, interleukin (IL)-6, and tumor necrosis factor (TNF)-α [19]. Adipose tissue is also an important source of circulating inflammatory factors, with visceral adipose tissue contributing more than subcutaneous fat. Additionally, several studies have evaluated the predictive role of BMI and WC in people with excess weight [20, 21]. Systemic circulating inflammatory factors regulate adhesion molecules and chemokines in vivo, prompting the infiltration of lymphocytes into liver tissues during homing activities, which contributes to the development of metabolically related fatty liver [19]. In addition, excess weight causes an imbalance in the regulation of immune cells in the body’s adipose tissue. The increased presence of inflammatory immune cells in adipose tissue elevates systemic inflammation, resulting in complications such as IR, hyperglycemia, dyslipidemia, cardiometabolic diseases and metabolic syndrome [22, 23]. Although various inflammatory indicators have been found to be associated with MAFLD in previous studies [17, 19], research integrating TyG and inflammatory markers through calculation has been relatively scarce [15]. This has, to some extent, hindered our ability to explore and compare different inflammatory markers. However, we have expanded our focus from physical measurement indicators to metabolic factors, representing a significant breakthrough. This suggests that there is still considerable potential in the study of inflammation and insulin resistance.

TyG is recognized as an indicator of IR, and a series of compound indicators have been derived. Studies have found that TyG can be used to predict type 2 diabetes, metabolic syndrome, cardiovascular events [24]. However, few studies have examined both TyG and inflammation, with the earliest attempts focusing on cancer patients and involving only hsCRP [15]. Studies have shown that the composition and distribution of body fat is closely related to IR. Dysfunctional obese adipose tissue develops into insulin resistance and chronic low-grade systemic inflammation through lipotoxicity [25]. Yang investigated TyG was an effective comprehensive indicator for predicting fat volume, density and distribution, and it was an important predictor of visceral obesity when combined with BMI [26]. Given the correlation between IR and obesity, studies have begun to explore whether combining the TyG index with obesity-related indices can enhance risk stratification. Due to differences in disease outcomes, database selection and study design, there is controversy over the merits of TyG and related indices. Ke found combining TyG index with BMI and WC did not further improve identification of diabetes risk in the elderly population [27]. Li suggested TyG-BMI outperformed TyG in predicting new-onset diabetes [28]. Zhang found that TyG combined with obesity indices were superior for identifying metabolic syndrome in males and females [29]. However, it is evident that previous studies have primarily focused on the connection between IR and indicators of obesity risk. In contrast, our research broadens the scope by incorporating metabolic indicators and conducts a comprehensive comparison with existing measures.

Few studies have integrated the TyG index and hs-CRP using standardized formulas, and existing results of the combination of body measurements such as WC and BMI are inconsistent, thus we conducted the study. Individuals with excess weight are at risk of metabolic-related diseases. However, regular exercise brings the possibility of reversing chronic inflammation, and people who exercise regularly often have greater treatment adherence and better clinical intervention outcomes [4, 30]. Our study found, compared with traditional TyG-WC, TyG-BMI, and TyG, TyG-hsCRP had a better risk stratification effect. These results again provide evidence for anti-inflammatory therapy, and are consistent with recommendations of new clinical anti-inflammatory treatments [14, 15]. Of course, there are numerous inflammatory indicators, and their relationships with diseases are complex [17, 19]. We believe other potential inflammatory markers may also offer clinical predictive value. By integrating IR with various inflammatory indicators and conducting comparative analyses within the same category, the research will become more comprehensive, which is the direction we intend to pursue. It is worth mentioning that the predictive value of TyG-WC is better than that of TyG in our study, indicating that the TyG index solely is far from satisfying the need to reflect the metabolic situation of the body.

There is no unified explanation for the difference between TyG-hsCRP and other metabolic indices. However, there are possible reasons. Firstly, the inflammatory response mediated by macrophages and fat cells stimulates the stress response of various systems in people with obesity, leading to new biochemical reaction based on the existing metabolism. Therefore, the comprehensive consideration of inflammation and IR offers a more accurate reflection of the body’s metabolic status [20]. Secondly, the TyG index itself is an indicator of the distribution and content of fat, while body measurement indicators still reflect body fat. Therefore, evaluation of a single dimension may not superimpose its evaluation effect [29]. Finally, the average age of the participants was around 56 years old, and metabolic conditions may have a greater effect than physical measures for middle-aged and elderly people.

One advantage of our study is that it is the first study to focus on individuals with excess weight who engage in regular physical activity, and we utilized a composite indicator of IR and inflammation to predict outcomes. Our sensitivity analysis is comprehensive and the comparative test of the predictive power is sufficient. But the study has some limitations. First, the data were mainly collected by questionnaires, including assessments of exercise type and recollections of exercise duration, which may introduce inaccuracies in the crowd selection. Secondly, due to the lag in imaging data, relying solely on ICD-10 codes for diagnosing MAFLD may result in the omission of positive cases during the follow-up period. Therefore, the interpretation of the absolute effect estimate should be approached with caution. In addition, the population is predominantly white, and a more robust analysis may require data from multiple regions and ethnicities.

## Conclusions

The TyG-hsCRP index can effectively predict the risk of MAFLD and all-cause mortality in physically active individuals with excess weight. This could help physicians with exercise guidance or clinical interventions.

## Supplementary Information

Below is the link to the electronic supplementary material.

Supplementary Material 1

## Data Availability

The data that support the findings of this study are available from the corresponding author upon reasonable request.

## Declarations

### Guarantors

Mengchen Zou is the guarantor of this work and, as such, had full access to all the data in the study and takes responsibility for the integrity of the data and the accuracy of the data analysis.

## References

[1] Zhang X, Ha S, Lau HC, Yu J (2023) Excess body weight: novel insights into its roles in obesity comorbidities. Semin Cancer Biol 92:16–27 36965839 10.1016/j.semcancer.2023.03.008

[2] Gadde KM, Martin CK, Berthoud HR, Heymsfield SB (2018) Obesity: pathophysiology and management. J Am Coll Cardiol 71(1):69–84 29301630 10.1016/j.jacc.2017.11.011 PMC7958889

[3] Tate AR, Rao GHR (2024) Inflammation: is it a Healer, Confounder, or a promoter of cardiometabolic risks? Biomolecules. 14(8):948 10.3390/biom14080948 PMC11352362 39199336

[4] Tucker WJ, Fegers-Wustrow I, Halle M et al (2022) Exercise for primary and secondary prevention of cardiovascular disease: JACC focus seminar 1/4. J Am Coll Cardiol 80(11):1091–1106 36075680 10.1016/j.jacc.2022.07.004

[5] Halle M, Papadakis M (2024) A new dawn of managing cardiovascular risk in obesity: the importance of combining lifestyle intervention and medication. Eur Heart J 45(13):1143–1145 38366823 10.1093/eurheartj/ehae091

[6] Sattar N, Neeland IJ, McGuire DK (2024) Obesity and cardiovascular disease: A new dawn. Circulation 149(21):1621–1623 38768272 10.1161/CIRCULATIONAHA.123.065485

[7] He D, Qiu Y, Yan M et al (2023) Associations of metabolic heterogeneity of obesity with frailty progression: results from two prospective cohorts. J Cachexia Sarcopenia Muscle 14(1):632–641 36575595 10.1002/jcsm.13169 PMC9891922

[8] Zhang H, Tang X, Hu D, Li G, Song G (2022) Transition patterns of metabolism-weight phenotypes over time: A longitudinal study using the multistate Markov model in China. Front Public Health 10:1026751 36589938 10.3389/fpubh.2022.1026751 PMC9799718

[9] Alemany M (2024) The metabolic Syndrome, a human disease. Int J Mol Sci 25(4):2251 38396928 10.3390/ijms25042251 PMC10888680

[10] Wang J, Yan S, Cui Y, Chen F, Piao M, Cui W (2022) The diagnostic and prognostic value of the Triglyceride-Glucose index in metabolic Dysfunction-Associated fatty liver disease (MAFLD): A systematic review and Meta-Analysis. Nutrients 14(23):4969 36500999 10.3390/nu14234969 PMC9741077

[11] Zhong Q, Zhou R, Huang YN et al (2024) Frailty and risk of metabolic dysfunction-associated steatotic liver disease and other chronic liver diseases. J Hepatol S0168–8278(24):02502–02509 10.1016/j.jhep.2024.08.024 39218228

[12] Cui C, Qi Y, Song J et al (2024) Comparison of triglyceride glucose index and modified triglyceride glucose indices in prediction of cardiovascular diseases in middle aged and older Chinese adults. Cardiovasc Diabetol 23(1):185 38812015 10.1186/s12933-024-02278-z PMC11138075

[13] Zhou Z, Liu Q, Zheng M et al (2024) Comparative study on the predictive value of TG/HDL-C, TyG and TyG-BMI indices for 5-year mortality in critically ill patients with chronic heart failure: a retrospective study. Cardiovasc Diabetol 23(1):213 38902757 10.1186/s12933-024-02308-w PMC11191322

[14] El Assar M, Álvarez-Bustos A, Sosa P, Angulo J, Rodríguez-Mañas L (2022) Effect of physical Activity/Exercise on oxidative stress and inflammation in muscle and vascular aging. Int J Mol Sci 23(15):8713 35955849 10.3390/ijms23158713 PMC9369066

[15] Ruan GT, Deng L, Xie HL et al (2024) Systemic inflammation and insulin resistance-related indicator predicts poor outcome in patients with cancer cachexia. Cancer Metab 12(1):3 38273418 10.1186/s40170-024-00332-8 PMC10809764

[16] Suren Garg S, Kushwaha K, Dubey R, Gupta J (2023) Association between obesity, inflammation and insulin resistance: insights into signaling pathways and therapeutic interventions. Diabetes Res Clin Pract 200:110691 37150407 10.1016/j.diabres.2023.110691

[17] Rohm TV, Meier DT, Olefsky JM, Donath MY (2022) Inflammation in obesity, diabetes, and related disorders. Immunity 55(1):31–55 35021057 10.1016/j.immuni.2021.12.013 PMC8773457

[18] Sandsdal RM, Juhl CR, Jensen SBK et al (2023) Combination of exercise and GLP-1 receptor agonist treatment reduces severity of metabolic syndrome, abdominal obesity, and inflammation: a randomized controlled trial. Cardiovasc Diabetol 22(1):41 36841762 10.1186/s12933-023-01765-z PMC9960425

[19] Bishop NC, Wadley AJ, Hamrouni M, Roberts MJ (2023) Inactivity and obesity: consequences for macrophage-mediated inflammation and the development of cardiometabolic disease. Proc Nutr Soc 82(1):13–21 35996926 10.1017/S0029665122002671

[20] Wu Y, Li D, Vermund SH (2024) Advantages and limitations of the body mass index (BMI) to assess adult obesity. Int J Environ Res Public Health 21(6):757 38929003 10.3390/ijerph21060757 PMC11204233

[21] Ross R, Neeland IJ, Yamashita S et al (2020) Waist circumference as a vital sign in clinical practice: a consensus statement from the IAS and ICCR working group on visceral obesity. Nat Rev Endocrinol 16(3):177–189 32020062 10.1038/s41574-019-0310-7 PMC7027970

[22] Liu R, Nikolajczyk BS (2019) Tissue immune cells fuel Obesity-Associated inflammation in adipose tissue and beyond. Front Immunol 10:1587 31379820 10.3389/fimmu.2019.01587 PMC6653202

[23] Schleh MW, Caslin HL, Garcia JN et al (2023) Metaflammation in obesity and its therapeutic targeting. Sci Transl Med 15(723):eadf9382 37992150 10.1126/scitranslmed.adf9382 PMC10847980

[24] Kurniawan LB (2024) Triglyceride-Glucose index as A biomarker of insulin Resistance, diabetes Mellitus, metabolic Syndrome, and cardiovascular disease: A review. EJIFCC 35(1):44–51 38706737 PMC11063788

[25] Yazıcı D, Demir SÇ, Sezer H (2024) Insulin Resistance, Obesity, and lipotoxicity. Adv Exp Med Biol 1460:391–430 39287860 10.1007/978-3-031-63657-8_14

[26] Yang Q, Xu H, Zhang H et al (2023) Serum triglyceride glucose index is a valuable predictor for visceral obesity in patients with type 2 diabetes: a cross-sectional study. Cardiovasc Diabetol 22(1):98 37120516 10.1186/s12933-023-01834-3 PMC10148999

[27] Ke P, Wu X, Xu M et al (2022) Comparison of obesity indices and triglyceride glucose-related parameters to predict type 2 diabetes mellitus among normal-weight elderly in China. Eat Weight Disord 27(3):1181–1191 34195936 10.1007/s40519-021-01238-w

[28] Li X, Sun M, Yang Y et al (2022) Predictive effect of triglyceride Glucose-Related Parameters, obesity Indices, and lipid ratios for diabetes in a Chinese population: A prospective cohort study. Front Endocrinol (Lausanne) 13:862919 35432185 10.3389/fendo.2022.862919 PMC9007200

[29] Zhang X, Zhang T, He S et al (2022) Association of metabolic syndrome with TyG index and TyG-related parameters in an urban Chinese population: a 15-year prospective study. Diabetol Metab Syndr 14(1):118 35706038 10.1186/s13098-022-00855-4 PMC9202163

[30] Soong RY, Low CE, Ong V et al (2025) Exercise interventions for Depression, Anxiety, and quality of life in older adults with cancer: A systematic review and Meta-Analysis. JAMA Netw Open 8(2):e2457859 39903465 10.1001/jamanetworkopen.2024.57859 PMC11795328

