# Supplementary material for: The close relationship between inflammation and insulin resistance: a comparative perspective from a new angle
Source: Acta Diabetol. 2025 Oct 15;63(3):423–34. doi: 10.1007/s00592-025-02596-y (PMC13046618; doi:10.1007/s00592-025-02596-y)
Supplement: Supplementary file 1 — Supplementary Material 1 [file 592_2025_2596_MOESM1_ESM.docx]

Supplementary File

The Close Relationship Between Inflammation and Insulin Resistance: A Comparative Perspective from a New Angle

Xiaoyan Wang^1^, Run Yang^1^, Jingxiang Li^1^,Yongqi Liang^2^, Chenxi Jin^1^, Yining Xu^1^, Xianbo Wu^2,*^, Mengchen Zou^1,*^

Xiaoyan Wang, Run Yang and Jingxiang Li contributed equally to this work.

Authors’ affiliation

^1^Department of Endocrinology and Metabolism, Nanfang Hospital, Southern Medical University, Guangzhou, China

^2^Department of Occupational Health and Medicine, School of Public Health, Southern Medical University, Guangzhou, China

Short Title: insulin resistance, inflammation and excess weight

*Corresponding Author

^1^Mengchen Zou, Email: [zoumc163@163.com](mailto:zoumc163@163.com)

Postal Address: Department of Endocrinology and Metabolism, Nanfang Hospital, Southern Medical University, 1838 Guangzhou Road North, Guangzhou 510515, China

ORCID:0000-0003-1409-4645

^2^Xianbo Wu, Email: [wuxb1010@smu.edu.cn](mailto:wuxb1010@smu.edu.cn)

Postal Address: Department of Endocrinology and Metabolism, Nanfang Hospital, Southern Medical University, 1838 Guangzhou Road North, Guangzhou 510515, China

ORCID:0000-0002-2706-9599

Keywords: excess weight, inflammation, insulin resistance, physically active

Contents：

Figures:

**Figure S1.** Flowchart of the study cohort in the UK Biobank database.

**Figure S2.**The proportional hazards assumption of the model, A): MAFLD, B):all-cause mortality.

**Figure S3.** Sex‐specific time‐dependent predictive capacity of TyG-hsCRP, TyG-WC, TyG-BMI, and TyG for MAFLD and all-cause mortality.

**Figure S4.** Age‐specific time‐dependent predictive capacity of TyG-hsCRP, TyG-WC, TyG-BMI, and TyG for MAFLD and all-cause mortality.

**Figure S5.** Interaction analysis of the association between TyG-hsCRP and MAFLD and all-cause mortality in the UK Biobank.

Tables:

**Table S1.** Details of cumulative dietary risk scores in the UK Biobank.

Participants from UK Biobank 2006-2010

N=502507

Participants without MAFLD or CVD events

N=86762

Participants with enthic smoke drink sleep data

N=72262

enthic smoke drink sleep data missing

Age under 20

N=2063

diagnosed with MAFLD or CVD events before recruitment

N=2690

Participants with triglycerides, glucose, hs-CRP HDL-C BMI waist

N=74325

Triglycerides, glucose, hs-CRP BMI waist data missing

N=12437

not meeting recommended physical activity or not overweight

N=413055

Participants meeting recommended physical activity and overweight

N=89452

Figure S1. Flowchart of the study cohort in the UK Biobank database

| A. | MAFLD | 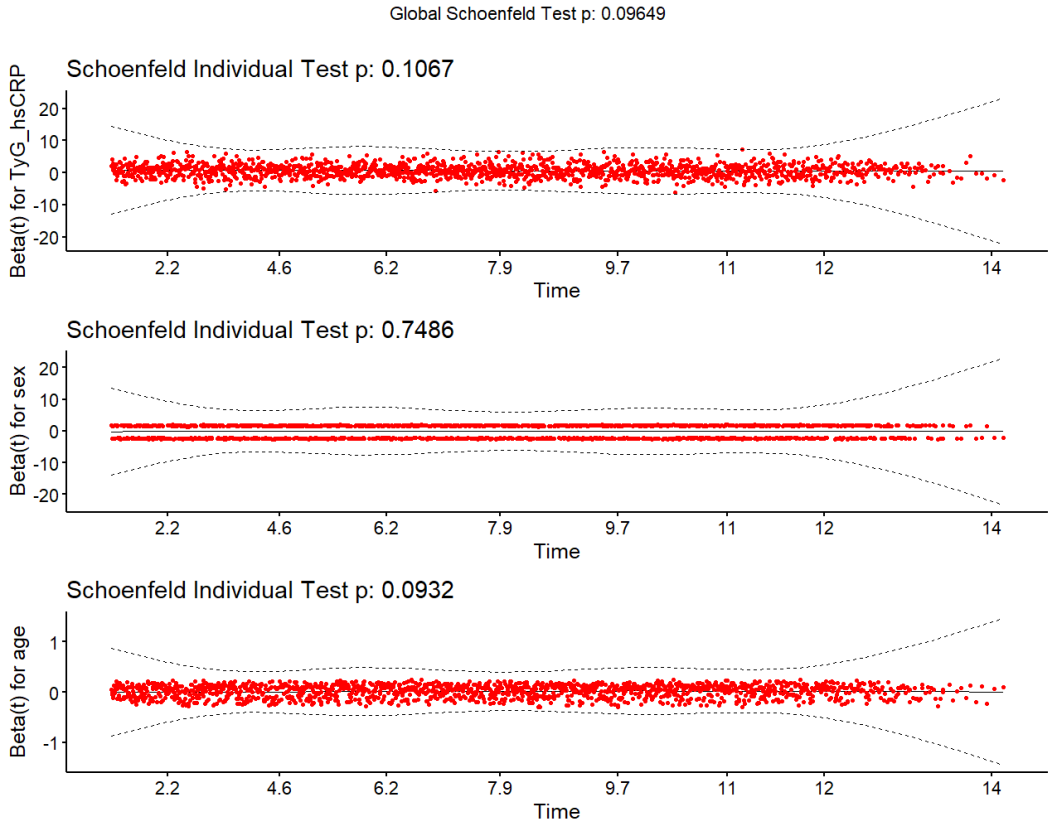 |
| --- | --- | --- |
| B. | all-cause mortality | 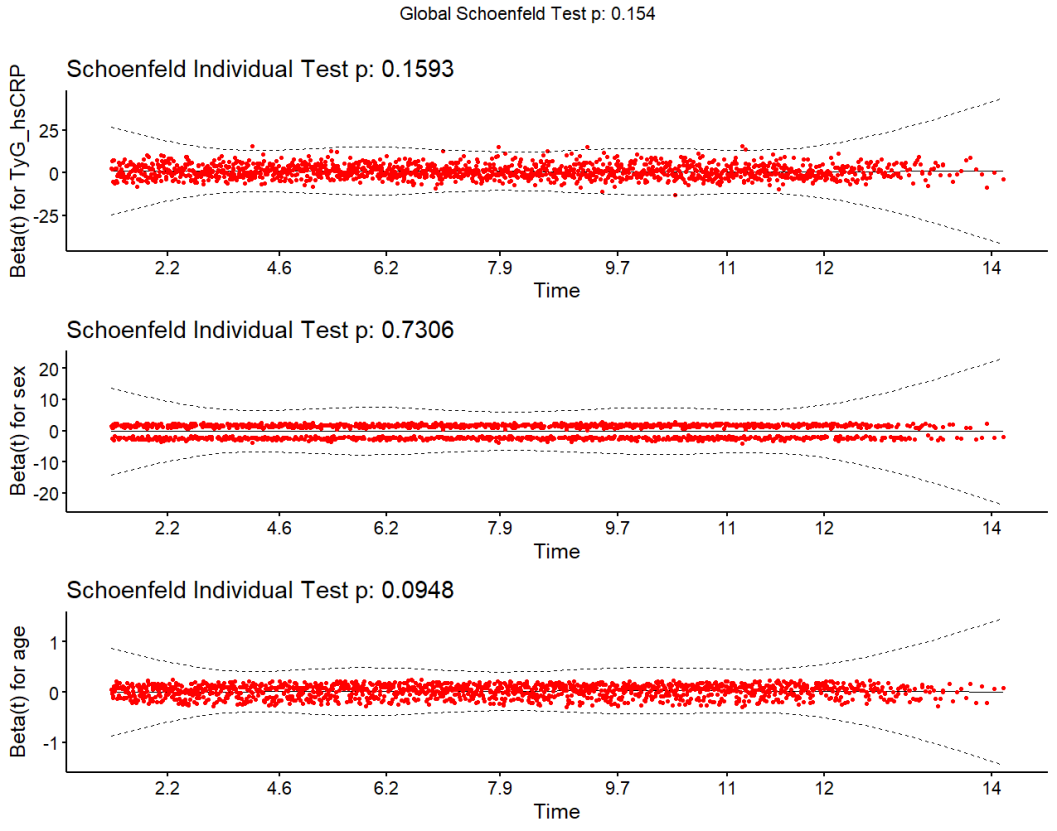 |

Figure S2.The proportional hazards assumption of the model, A): MAFLD, B):all-cause mortality.

|  |  | Female | Male |
| --- | --- | --- | --- |
| A. | MAFLD | 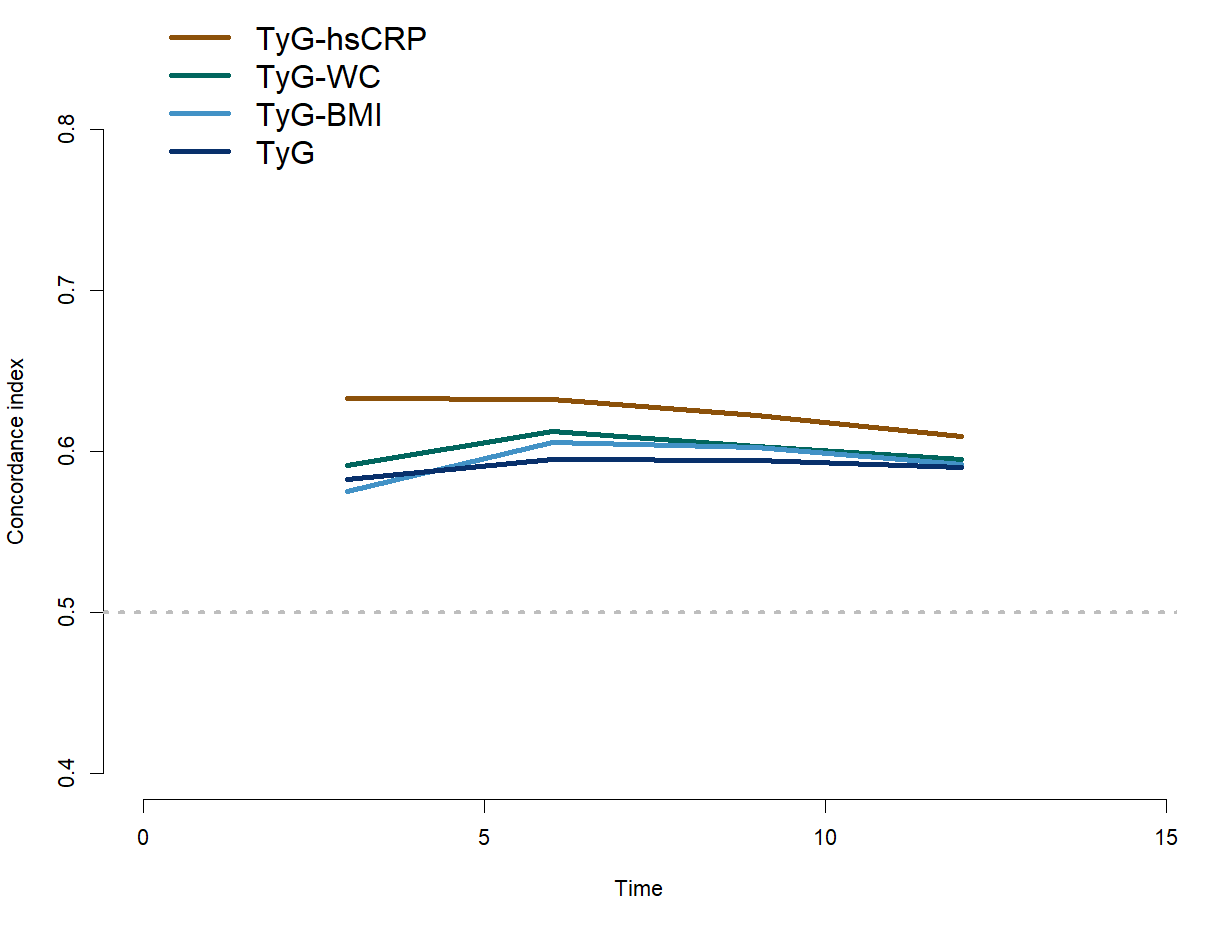 | 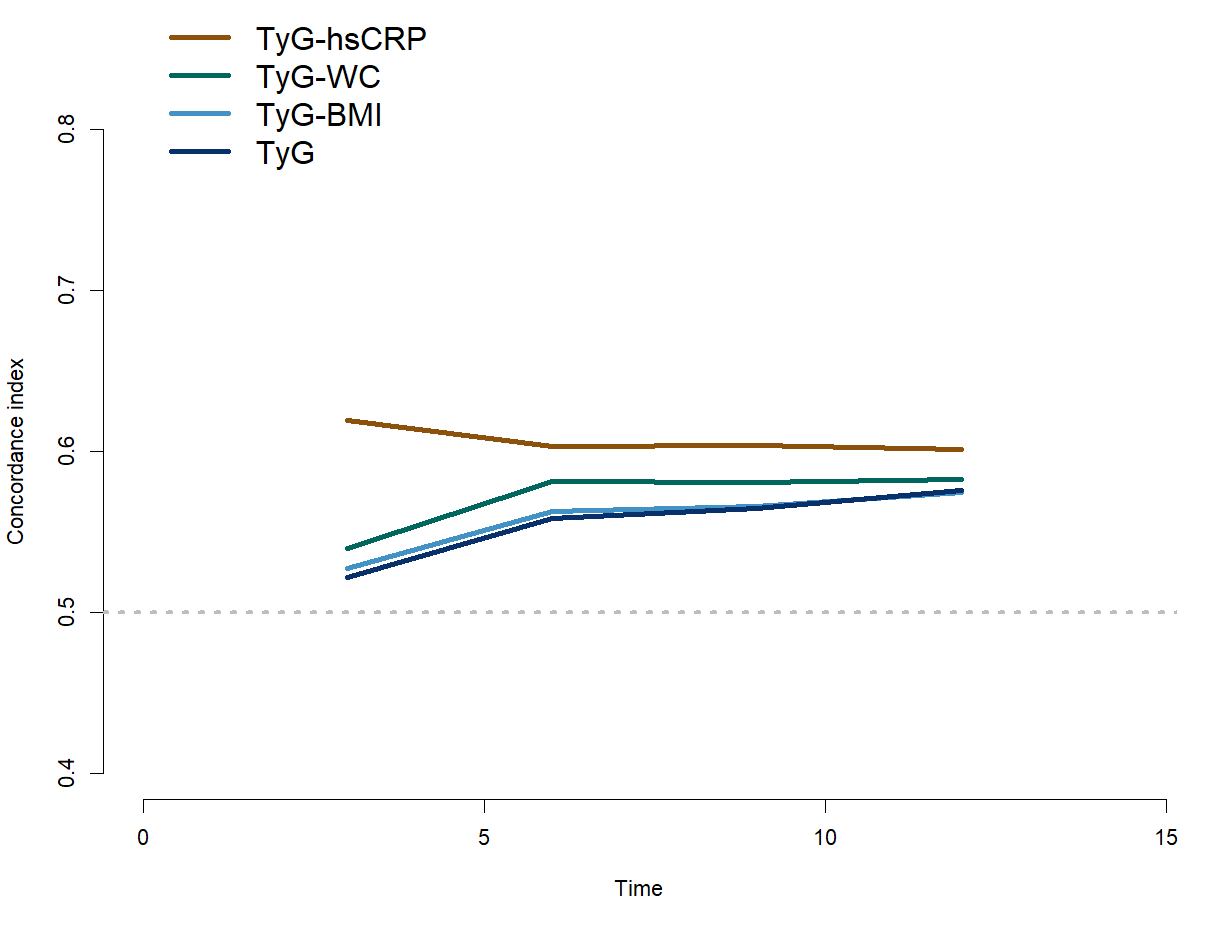 |
| B. | all-cause mortality | 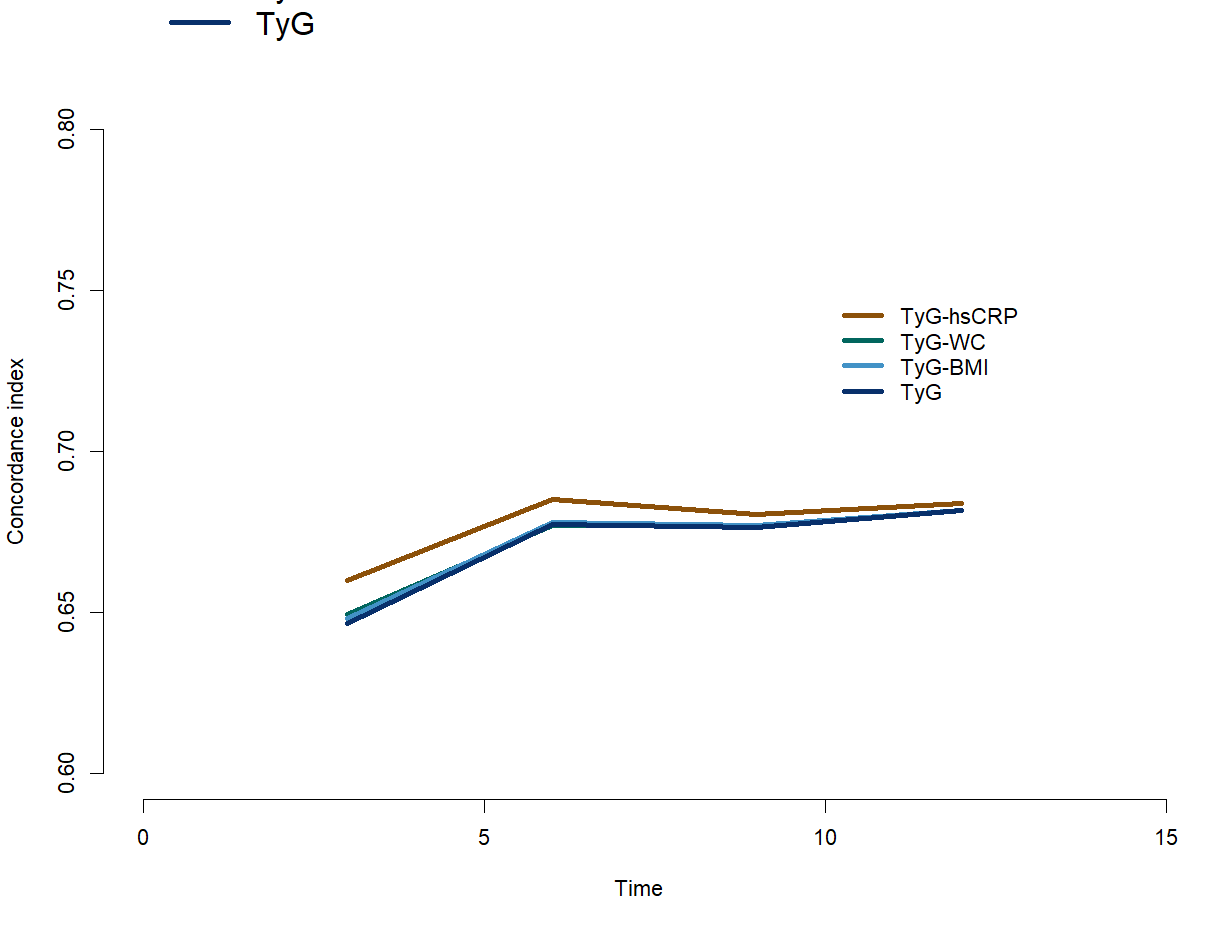 | 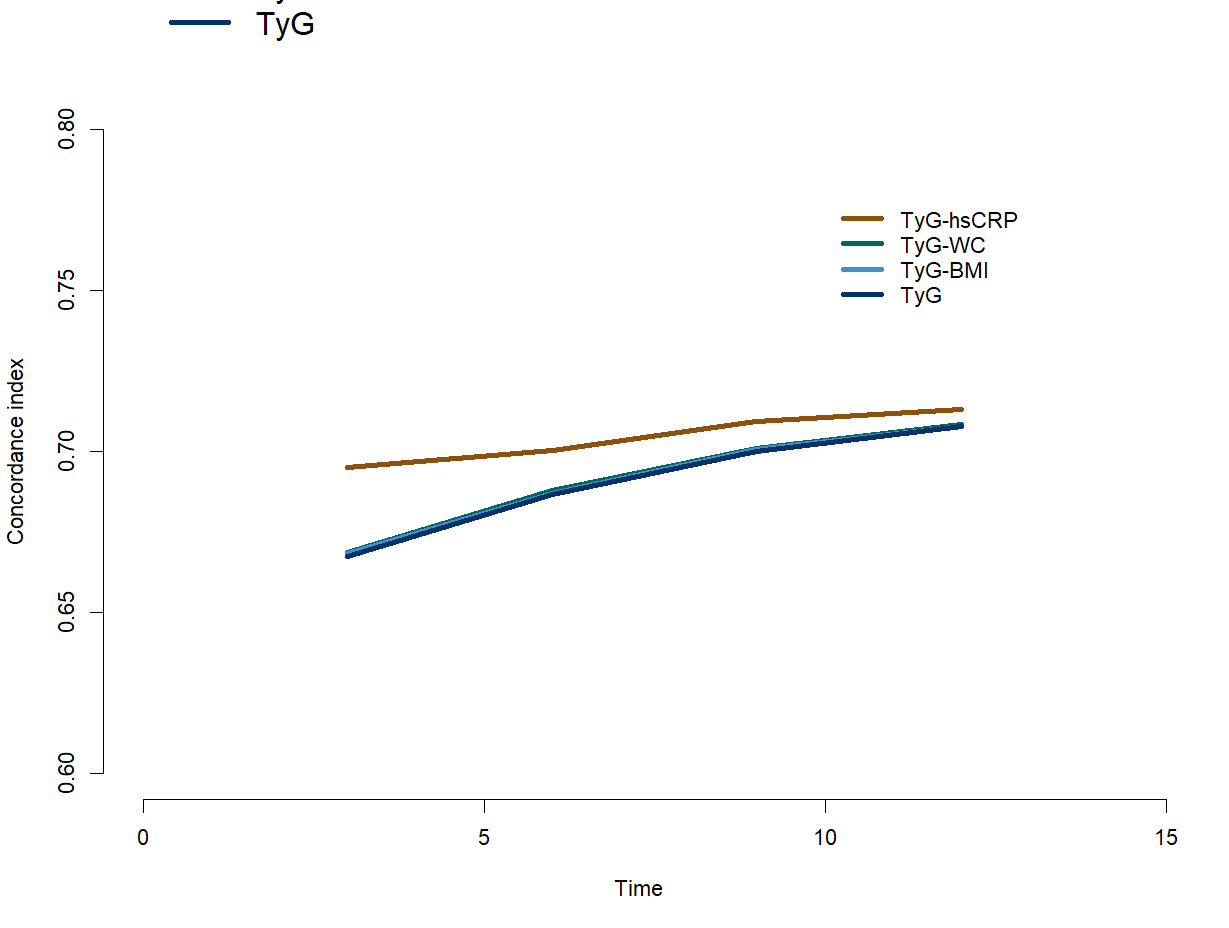 |

Figure S3: Sex‐specific time‐dependent predictive capacity of TyG-hsCRP, TyG-WC, TyG-BMI, and TyG for MAFLD (A) and all-cause mortality (B). Figure legends: Brown lines = group TyG-hsCRP; Green lines =group TyG-WC; Light blue lines = group TyG-BMI; Dark blue line = group TyG.

|  |  | Age:20-60 | Age:>60 |
| --- | --- | --- | --- |
| A. | MALLD | 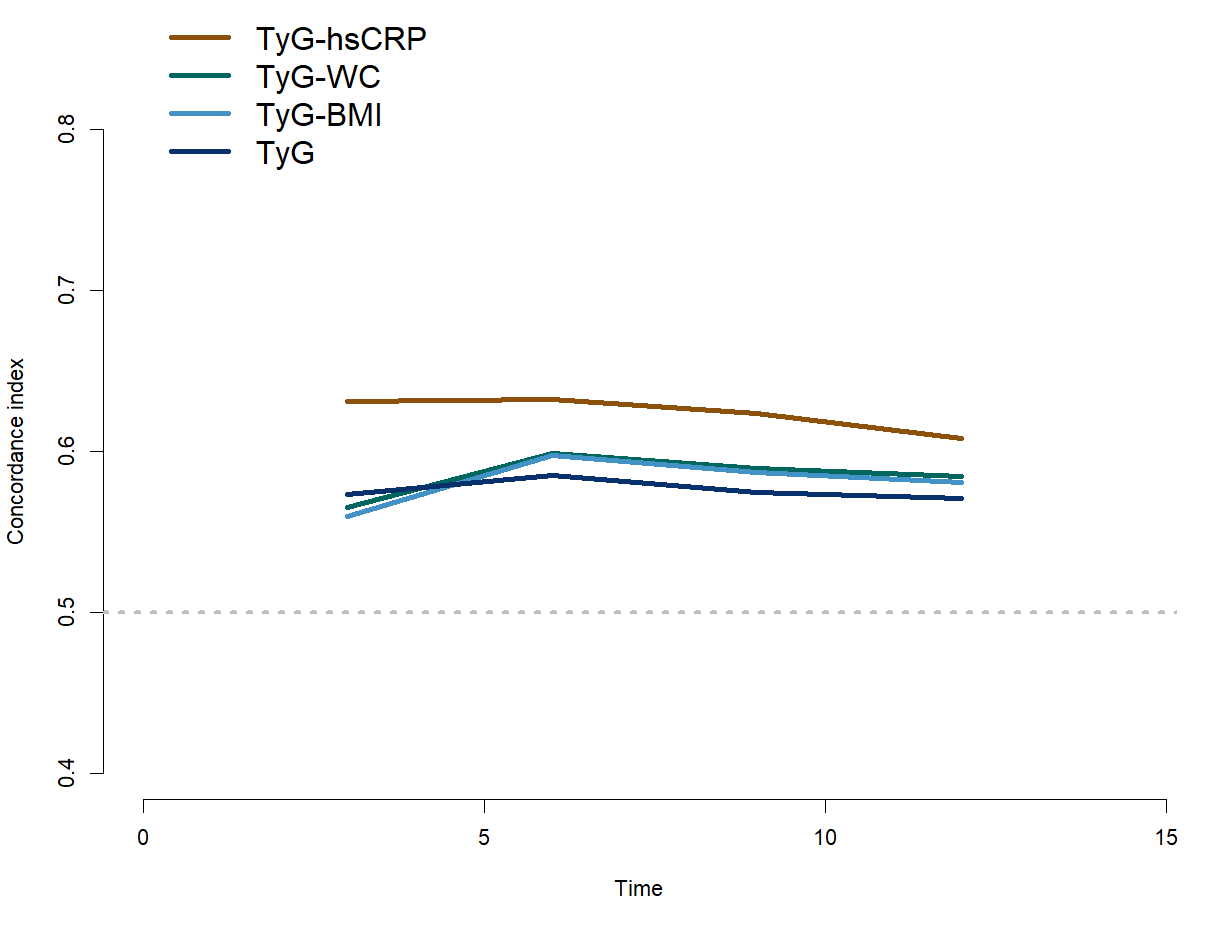 | 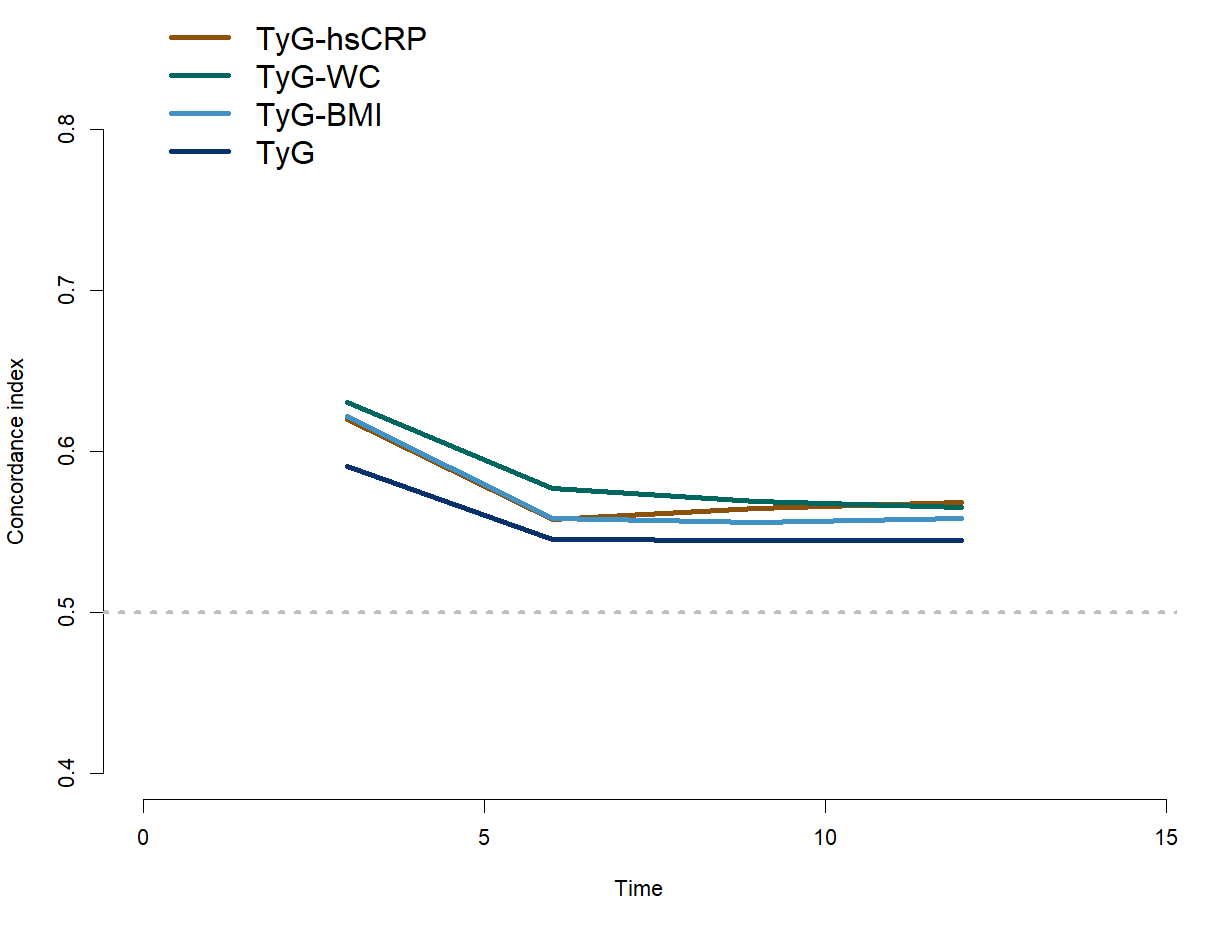 |
| B. | All-cause mortality | 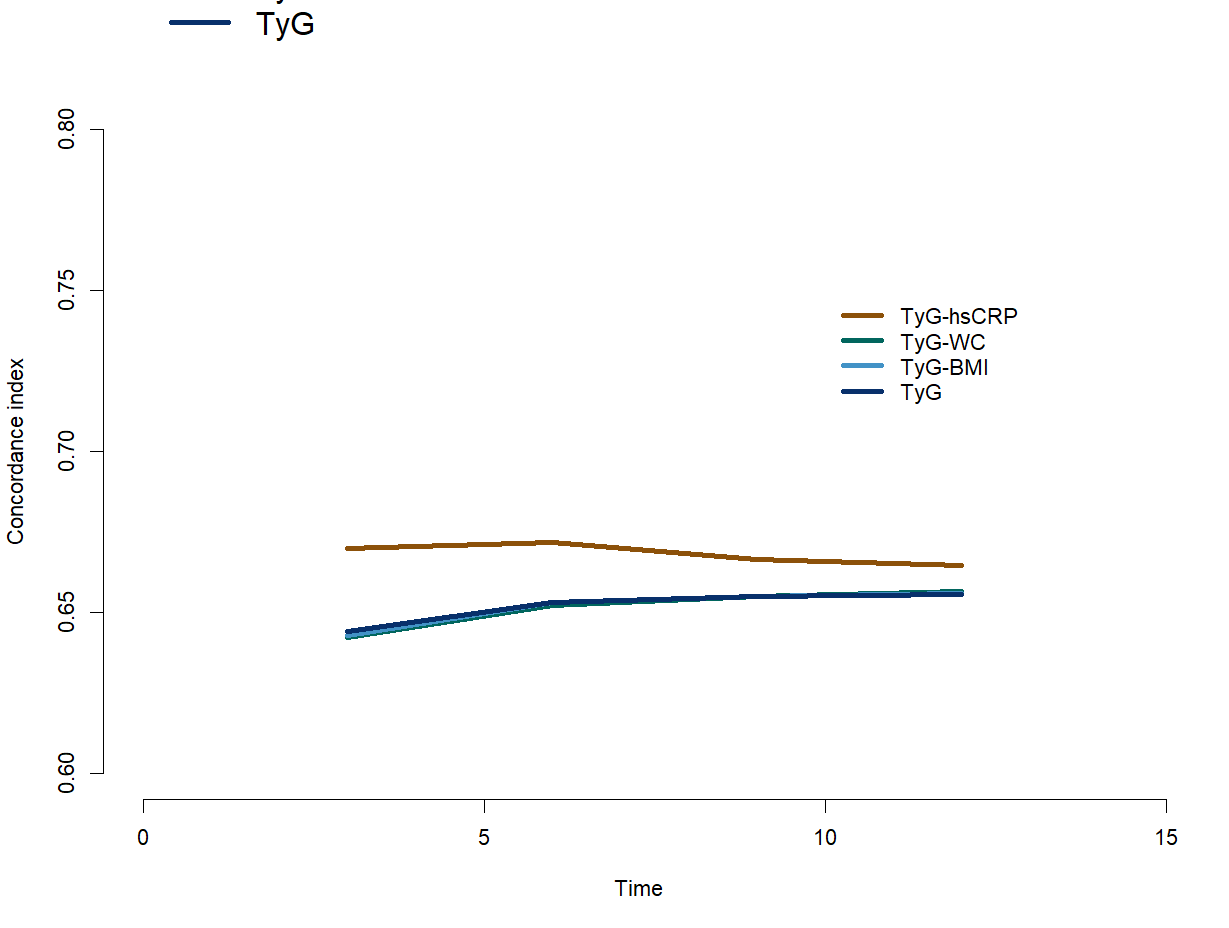 | 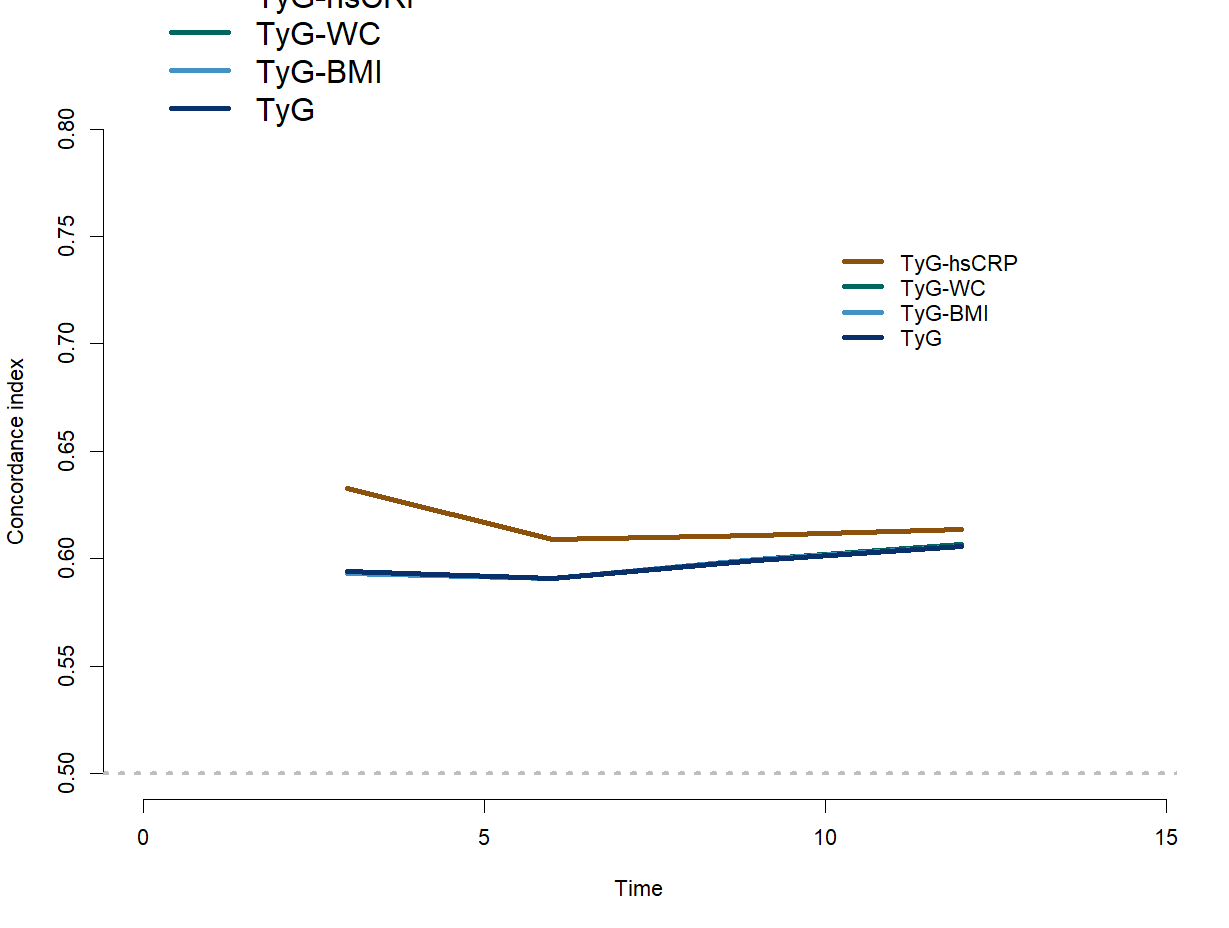 |

Figure S4: Age‐specific time‐dependent predictive capacity of TyG-hsCRP, TyG-WC, TyG-BMI, and TyG for MAFLD (A) and all-cause mortality (B). Figure legends: Brown lines = group TyG-hsCRP; Green lines =group TyG-WC; Light blue lines = group TyG-BMI; Dark blue line = group TyG.

| A. | MAFLD | 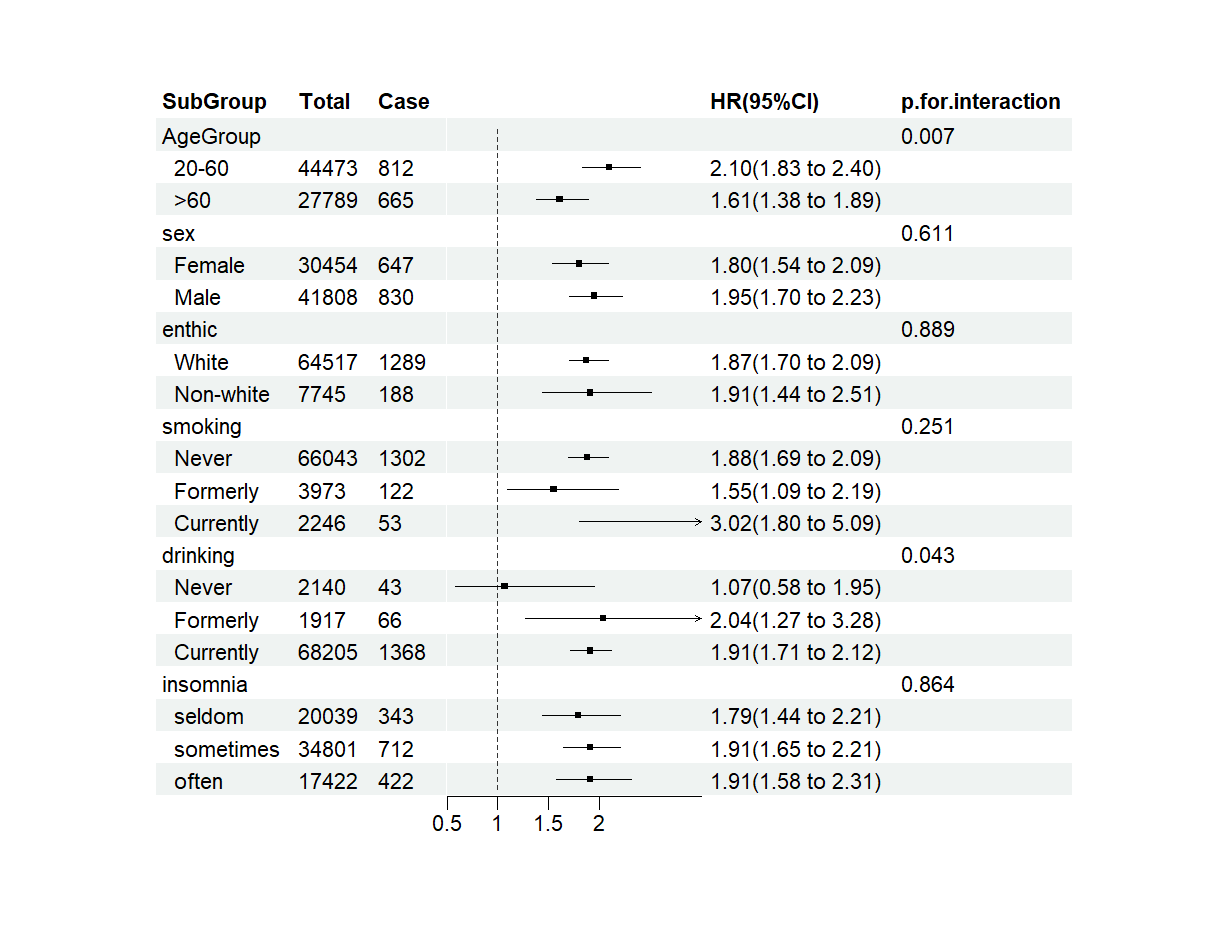 |
| --- | --- | --- |
| B. | all-cause mortality | 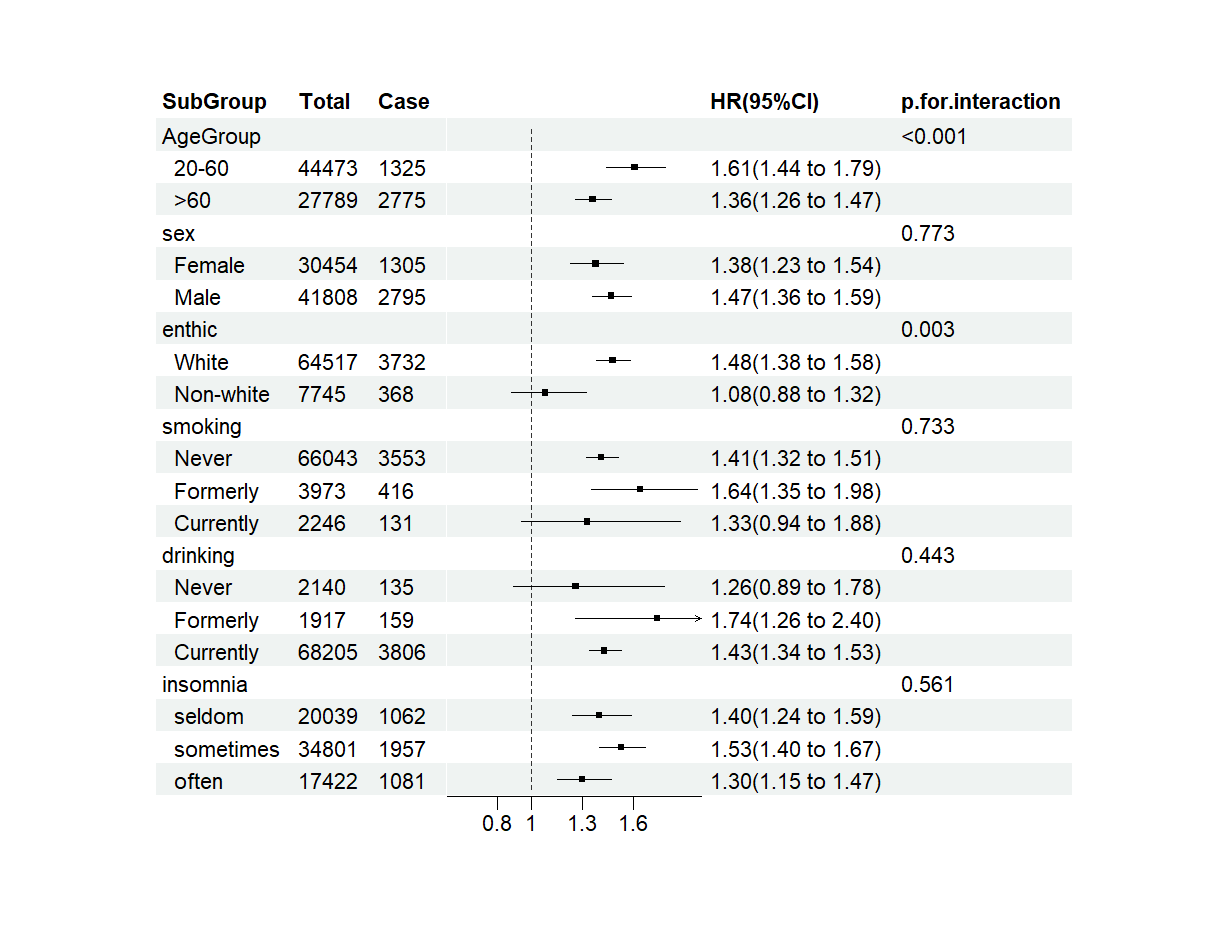 |

Figure S5: Interaction analysis of the association between TyG-hsCRP and MAFLD (A) and all-cause mortality (B) in the UK Biobank.

Table S1. Details of cumulative dietary risk scores in the UK Biobank.

| Variable | Variable | Unit conversion | Binary Variables: |
| --- | --- | --- | --- |
| Fruit & vegetables (regrouped from fruit, dried fruit & Vegetable) | Fresh fruit intake (pieces/day) | Amount per serving: 1 piece 0.5 Less than one | 0 > = 5 serving/day (Ref.)  1 <5 serving/day |
|  | Dried fruit intake (pieces/day) | Amount per serving: 2 piece 0.5 Less than one |  |
|  | Cooked vegetable intake (tablespoons/day) | Amount per serving: 2 heaped tablespoons 0.5 Less than one |  |
|  | Salad / raw vegetable intake (tablespoons/day) | Amount per serving: 2 heaped tablespoons 0.5 Less than one |  |
| Total fish intake (regrouped from Both total non-oily fish and oily fish) | Oily fish intake (per week) | 0.5 Less than one 1 Once a week 3 2-4 times a week 5.5 5-6 times a week 7 Once or more daily | 0 > = 2 times a week (at least once a week of each category) (Ref.) 1 < once a week of each one |
|  | Non-oily fish intake (per week) | 0.5 Less than one 1 Once a week 3 2-4 times a week 5.5 5-6 times a week 7 Once or more daily |  |
| Processed meat intake | Processed meat intake (per week) | 0.5 Less than one 1 Once a week 3 2-4 times a week 5.5 5-6 times a week 7 Once or more daily | 0 < = Once a week (Ref.) 1 > Once a week |
| Red meat (regrouped from beef, pork and lamb) | Beef intake (per week) | 0.5 Less than one 1 Once a week 3 2-4 times a week 5.5 5-6 times a week 7 Once or more daily | 0 < = Once a week (Ref.) 1 >Once a week |
|  | Lamb/mutton intake (per week) | 0.5 Less than one 1 Once a week 3 2-4 times a week 5.5 5-6 times a week 7 Once or more daily |  |
|  | Pork intake (per week) | 0.5 Less than one 1 Once a week 3 2-4 times a week 5.5 5-6 times a week 7 Once or more daily |  |
| Milk type used | Milk type used |  | 0 Semi-skimmed/skimmed (Ref.)  1 Full cream/ another type of milk/ never rarely have milk |
| Spread type | Spread type |  | 0 Never/rarely (Ref.)  1 Another selection |
| Cereal intake * | Cereal intake (Bowls/week ) | Amount per serving: Bran/oat/muesli cereal– 1 bowl/day 0.5 Less than one | 0 >5 bowls (Ref)  1 < = 5 bowls |
| Salt added to food | Salt added to food |  | 0 Never/rarely (Ref.)  1 Another selection |
| Water intake | Water intake (Glasses/day) |  | 0 > = 6 glasses (Ref.)  1 <6 glasses |
